# Risk Factors for Human Papillomavirus Positivity in a Tertiary Care Center: A Case–Control Study Incorporating Genotype Distribution, Co-Infection Patterns, and Quantitative Viral Load Analysis

**DOI:** 10.3390/jcm15166162

**Published:** 2026-08-08

**Authors:** Mete Hakan Karalök, Bağnu Dündar, Ayhan Parmaksız, Asiye Gök Yurttaş

**Affiliations:** 1 Department of Obstetrics and Gynaecology, Faculty of Medicine, Istanbul Atlas University, Istanbul 34408, Türkiye; hakan.karalok@atlas.edu.tr; 2 Department of Medical Biochemistry, Faculty of Medicine, Istanbul Atlas University, Istanbul 34408, Türkiye; bagnu.dundar@atlas.edu.tr; 3 Department of Biostatistics, Faculty of Medicine, Istanbul Health and Technology University, Istanbul 34275, Türkiye; ayhan.parmaksiz@istun.edu.tr

**Keywords:** human papillomavirus, viral copy number, coinfection, tertiary care center, anogenital warts

## Abstract

**Background/Objectives**: Human papillomavirus (HPV) is the leading cause of cervical cancer and anogenital malignancies, yet its clinical presentation, genotype distribution, co-infection patterns, and viral load are poorly characterised in tertiary-care referrals. This study aimed to identify predictors of HPV positivity and describe genotype, co-infection, and relative viral copy number (Cq values) in this setting. **Methods**: A retrospective case–control study of 234 patients (97 HPV-positive, 137 HPV-negative) used a 37-genotype qPCR platform at a tertiary-care hospital between January-December 2025. Demographic data, clinical diagnosis categories, genotype profiles, co-infection patterns, and cycle quantification (Cq) values were recorded, and multivariable logistic regression identified independent predictors of HPV positivity. **Results**: HPV positivity was 41.5% (97/234). Only the clinical diagnosis category independently predicted HPV status. Compared with non-specific presentations, patients with anogenital or viral warts (aOR: 4.25; 95% CI: 1.49–12.14; *p* = 0.007) and vaginal or vulvar inflammation (aOR: 2.08; 95% CI: 1.19–3.63; *p* = 0.010) had higher odds of positivity. High-risk genotypes were the second most frequently detected category (40.00% of detections), after low-risk genotypes (48.21%), with HPV-16 leading among high-risk types. Co-infection occurred in 44.3% of HPV-positive patients, mostly involving mixed-risk genotypes. Patients with anogenital warts had lower Cq values than those with urinary tract complaints (*p* = 0.011), reflecting higher relative viral copy numbers per swab. **Conclusions**: HPV positivity and relative viral copy number (as approximated by Cq values) tracked more closely with clinical presentation than with demographic background. The dominance of low-risk and high-risk genotypes and the prevalence of mixed-risk co-infections were consistent with the symptomatic profile of the cohort. These findings support interpreting HPV results in light of clinical presentation and extended genotyping with relative viral copy number quantification in tertiary settings.

## 1. Introduction

Human papillomavirus (HPV) is the most common sexually transmitted infection worldwide and the principal etiological agent of cervical cancer, as well as a driver of anogenital and oropharyngeal malignancies [1,2]. Globally, cervical cancer remains the fourth most common cancer in women, accounting for approximately 660,000 new cases and 350,000 deaths in 2022, with 94% of those deaths occurring in low- and middle-income countries [3]. Oncogenic transformation is mediated by the viral E6 and E7 proteins, which degrade the tumor suppressors p53 and pRb, respectively, thereby driving uncontrolled cellular proliferation and immune evasion [4]. Persistent infection with high-risk HPV genotypes, rather than transient exposure, is considered the key prerequisite for cervical carcinogenesis, a distinction that renders genotype identification and viral persistence central to clinical risk assessment.

The likelihood of HPV positivity in a clinical population is shaped by a combination of demographic and clinical factors. Age is among the most consistently reported predictors, with prevalence peaking in younger sexually active individuals and showing variable patterns in older age groups depending on the population studied [5]. Furthermore, while clinical focus has historically centered on women, HPV remains a significant contributor to urological and anogenital pathologies in men, necessitating gender-inclusive epidemiological data [6]. Gender and geographic origin further modulate exposure patterns, reflecting differences in sexual health behaviors, screening uptake, and HPV vaccination coverage across populations [7]. Critically, the clinical diagnosis at presentation, whether a patient attends for gynecological screening, inflammatory conditions, anogenital lesions, or urological complaints, is a strong contextual determinant of HPV test positivity, yet its independent contribution is rarely quantified in controlled study designs. These gaps highlight the need for case–control studies that examine demographic and clinical predictors of HPV positivity together, within a real-world tertiary care setting. Most published case–control studies from the region have focused either on genotype-level prevalence or on demographic risk factors in isolation, without simultaneously adjusting for both. We therefore hypothesised that clinical diagnosis category at the time of testing would independently predict HPV positivity, over and above age, sex, and geographic origin, within a tertiary-care population.

Prior single-centre studies from Türkiye have documented HPV genotype frequencies, predominantly in female screening populations, but have rarely extended to mixed-sex tertiary-care cohorts or integrated quantitative viral load data alongside prevalence estimates [8,9,10,11]. Genotype-specific differences in oncogenic potential make local surveillance data indispensable for clinical decision-making. High-risk genotypes, particularly HPV-16 and HPV-18, drive the overwhelming majority of cervical malignancies, while low-risk types are associated with benign lesions. In Türkiye, HPV-16 has been consistently identified as the most prevalent high-risk genotype across multiple study populations, followed by HPV-18, -31, -45, and -51 [8,9,10]. A 2025 cross-sectional study from Ankara further documented an HPV positivity rate of 18.2% among unvaccinated women, with 28.6% of positive cases involving multiple genotypes, underscoring the heterogeneous distribution of HPV types within Türkiye and the importance of extended genotyping in clinical practice [11].

Building on studies that identified co-infection as a potential modifier of disease progression [12], the present study examines whether genotype co-occurrence patterns in a symptomatic tertiary-care setting differ from those reported in organised screening programmes. Co-infection with multiple HPV genotypes adds a further dimension of clinical complexity. Whether simultaneous infection with more than one genotype amplifies or attenuates disease progression remains contested: some studies report a synergistic effect on high-grade squamous intraepithelial lesion (HSIL) development, while others suggest intergenotypic competition may limit pathogenicity. Indeed, across large screening populations, multiple-genotype infections account for a substantial proportion of HPV-positive cases, often ranging between 20% and 30%, with HPV-16 being the most frequently co-detected type [12]. Resolving this ambiguity requires population-specific data that characterize co-infection patterns alongside clinical outcomes.

Liu et al. [13] and Varesano et al. [14] established associations between HPV viral load and cervical lesion severity; whether these associations hold across the broader clinical spectrum of a tertiary-care referral population has not been examined. Quantitative viral load analysis has emerged as a clinically relevant complement to genotyping. Quantitative PCR-based platforms express viral burden through cycle quantification (Cq) values, where lower Cq values correspond to higher viral copy numbers. Higher viral loads have been associated with persistent infection, reduced likelihood of spontaneous clearance, and elevated risk for high-grade cervical lesions [13]. A 2025 retrospective study integrating viral load and co-infection data in colposcopy patients confirmed that both markers were significantly more frequent in high-grade CIN, supporting their combined use in screening triage [14]. Nevertheless, the prognostic significance of viral load remains debated across genotypes and populations, warranting further investigation in settings where quantitative data are already routinely generated. Moreover, the presence of clinically HPV-associated lesions in molecularly negative cases which are potentially due to low viral burden or sampling limitations, and temporal fluctuations in testing can provide critical context for assessing routine diagnostic performance [15].

Despite this growing body of evidence, several important gaps remain. First, existing studies from Türkiye have largely reported HPV prevalence or genotype frequencies in isolation, without simultaneously controlling for demographic and clinical covariates in a case–control framework [8,9,10,11]; as a result, the independent contribution of clinical presentation to HPV test positivity, as distinct from age, sex, or geographic origin, has not been formally quantified. Second, co-infection prevalence data from the region derive predominantly from cervical screening cohorts, leaving the co-infection landscape of mixed-sex symptomatic tertiary-care populations largely uncharacterised [12]. Third, although quantitative viral load has been associated with lesion severity and persistence in colposcopy settings [13,14], its relationship with genotype risk category, clinical diagnosis, and co-infection status within a single integrated dataset has not been reported from Türkiye. Finally, existing studies have rarely employed broad-spectrum genotyping platforms capable of detecting low-risk and probable/possible high-risk (pHR-HPV; IARC Group 2A/2B) genotypes alongside carcinogenic high-risk types.

The present retrospective case–control study was therefore designed to address these gaps simultaneously. Specifically, we aimed to: (i) identify independent clinical and demographic predictors of HPV positivity using multivariable logistic regression; (ii) characterise the full genotype distribution and risk category profile of a mixed-sex tertiary-care cohort using a 37-genotype quantitative PCR platform; (iii) describe co-infection patterns and assess whether co-infection is associated with higher relative viral copy number; and (iv) explore temporal distribution patterns across a 12-month observation window. We hypothesised that clinical diagnosis category at the time of presentation would independently predict HPV positivity beyond conventional demographic risk factors, and that Cq values, would vary by genotype risk category and clinical presentation rather than by patient demographics. Given that national HPV vaccination coverage in Türkiye remains suboptimal [16], findings from clinically grounded epidemiological studies such as this one carry direct implications for local screening strategies, genotype-specific surveillance, and preventive policy.

## 2. Materials and Methods

### 2.1. Study Design and Setting

This retrospective case–control study was conducted at Istanbul Atlas University Hospital, a tertiary care referral centre, over a 12-month period spanning January to December 2025. The study aimed to identify clinical and demographic predictors of HPV positivity, characterise HPV genotype distribution and co-infection patterns, and evaluate relative viral copy number (approximated by Cq values) in relation to genotype risk category and clinical presentation. Ethical approval was obtained from the institutional review board prior to data extraction, and all analyses were performed in accordance with the principles of the Declaration of Helsinki. Patient data were anonymised at the point of collection, and no direct patient contact was required.

### 2.2. Study Population

The study population comprised all patients who underwent HPV nucleic acid testing (NAT) at the study centre during the 12-month study period. Eligibility was assessed against pre-specified inclusion and exclusion criteria.

Inclusion criteria were as follows: HPV NAT performed between January and December 2025 at Istanbul Atlas University Hospital; age 18 years or older at the time of testing; and complete demographic and clinical records available in the hospital information management system (HIMS).

The following exclusion criteria were applied: age below 18 years; active pregnancy documented in the medical record where HPV testing was performed for obstetric indication; prior history of HPV-directed treatment (ablation, loop electrosurgical excision procedure, or equivalent); patients who underwent more than one HPV test within the same calendar year (only the first test result was retained); and patients with missing demographic data (age or sex) or clinical diagnosis.

Cases were defined as patients with a positive HPV NAT result (*n* = 97). Controls were patients tested during the same period at the same institution who received a negative HPV result, selected to reflect a comparable clinical referral profile (*n* = 137). Controls were not individually matched to cases; instead, consecutive HPV-negative patients tested during the same 12-month period at the same institution were enrolled to ensure a comparable clinical referral context. No restriction was applied on the basis of age, sex, or diagnosis at the time of enrolment, as these variables were treated as potential predictors in the regression analyses. The final analytic cohort comprised 234 participants.

### 2.3. Sample Size and Power Analysis

Sample size estimation was based on HPV positivity rates reported from Turkish tertiary care settings, which range from approximately 35% to 40%. Using a two-group proportion comparison with a significance level of 0.05 (two-tailed), a power of 80%, and a case-to-control ratio of approximately 1:1.4, the target sample was determined using G*Power software (version 3.1, University of Dusseldorf, Germany). The final sample of 97 cases and 137 controls satisfied these requirements. For the multivariable logistic regression model, application of the events-per-variable rule (EPV = 10) confirmed that the case group size of 97 would support the safe inclusion of up to nine predictor variables without model overfitting. Subgroup analyses conducted within restricted strata (for example, male patients only; *n* = 16) are presented as exploratory rather than confirmatory, given reduced statistical power in these subsets.

### 2.4. Data Collection

Data were extracted retrospectively from two institutional systems: the hospital information management system (HIMS) and the laboratory information system (LIS). The extraction procedure was performed by authorised members of the research team in two sequential stages. In the first stage, all patients who underwent HPV NAT between January and December 2025 were identified through the LIS, and eligibility screening was applied to generate the study list. In the second stage, demographic information (age, sex, geographic origin), clinical diagnosis at the time of testing, test date (month and year), and HPV test results were retrieved from the HIMS for each eligible patient. All personal identifiers (name, national identification number, and contact details) were removed prior to data entry, and each participant was assigned a sequential anonymous study number. Anonymised data were stored in an encrypted electronic file accessible only to the research team.

For each HPV-positive patient, the genotype-level test report was also retrieved from the LIS. This report specified the HPV genotype or genotypes detected and the corresponding Cq value for each positive genotype. A standardised data extraction form was used to transfer these results into a structured electronic table. Patients with multiple genotypes contributed one row per detected genotype in the long-format dataset used for genotype-level analyses, while patient-level analyses retained one row per patient.

### 2.5. HPV Testing Methodology

Cervical, vaginal, and penile swab samples were collected by trained clinical staff in accordance with national and international specimen collection guidelines. Samples were transported to the accredited microbiology laboratory in vNAT^®^ Transfer Tubes (Cat. No: BS-NA-513m-100, Bioeksen AR-GE Teknolojileri A.Ş., Istanbul, Türkiye) and stored at +2 °C to +8 °C for up to three months prior to processing. HPV nucleic acid testing was performed using the Bio-Speedy^®^ GHPV-L Genotyping qPCR Panel (Bioeksen AR-GE Teknolojileri A.Ş., Istanbul, Türkiye; Cat. No: BS-GHPV-L-25/BS-GHPV-L-100), a real-time quantitative PCR assay capable of simultaneously detecting 37 distinct HPV genotypes plus an internal human control. Nucleic acid extraction was performed using the Zybio EXM3000 automated extraction system (Zybio Inc., Chongqing, China) with the BioSpeedy^®^ Rapid Nucleic Acid Extraction Kit (Cat. No: ZFNAE01, Bioeksen AR-GE Teknolojileri A.Ş.). Amplification and Cq determination were carried out on a qPCR Device [BMS Magnetic Induction Cycler (Mic) (Bio Molecular Systems, Upper Coomera, QLD, Australia; S/N: M0007127)] using the following thermal cycling programme: enzyme activation at 52 °C for 3 min; pre-heating at 95 °C for 10 s; 12 touchdown denaturation cycles (denaturation at 95 °C for 1 s; annealing/extension from 67 °C decreasing to 56 °C at 1 °C per cycle for 15 s); followed by 30 amplification cycles (denaturation at 95 °C for 1 s; annealing/extension at 55 °C for 15 s). Fluorescence was detected in FAM, HEX, ROX, and CY5 channels. According to the manufacturer’s validation data, the assay demonstrates a sensitivity of 99.50% and specificity of 98.48%. The Cq value corresponds to the number of PCR amplification cycles required to reach a defined fluorescence threshold; it is inversely proportional to the initial viral copy number in the sample, such that a lower Cq value indicates a higher viral copy number per swab. In the present study, Cq values were used as a surrogate measure of quantitative viral copy number; these values are not normalised to cellular DNA and should not be interpreted as absolute intracellular viral burden.

A genotype was reported as positive when the amplification curve was sigmoidal and the Cq value fell below the genotype-specific threshold at a relative fluorescence unit (RFU) cut-off of 0.5 (0.2 for the internal human control). The Cq limit was ≤30 for most genotypes and ≤26 for HPV-18 and HPV-35. Non-sigmoidal amplification curves were reported as negative regardless of Cq value, per the manufacturer’s interpretation guidelines.

Each analytical run incorporated a negative template control (NTC) to monitor inter-run contamination and a positive control (PC Mix) to verify reagent performance and amplification efficiency. An internal human control (IC) was included in every reaction well to confirm adequate nucleic acid extraction and sample quality. Runs yielding invalid control results were excluded from analysis and repeated.

### 2.6. HPV Genotype Risk Classification

HPV genotypes detected in this study were classified into three risk categories in accordance with the carcinogenic potential framework established by the International Agency for Research on Cancer (IARC) [17]. High-risk genotypes (HR-HPV; IARC Group 1, carcinogenic to humans) comprised HPV types 16, 18, 31, 33, 35, 39, 45, 51, 52, 56, 58, 59, 66, and 68. Probable and possible high-risk genotypes (pHR-HPV; IARC Groups 2A/2B) comprised HPV types 53, 67, 70, 73, and 82. All remaining detected genotypes, including HPV types 6, 11, 40, 42, 43, 44, 54, 61, 62, 69, 71, 72, 74, 81, 83, 84, and 91, were classified as low-risk (LR-HPV; IARC Group 3, not classifiable as carcinogenic to humans). For patient-level risk categorisation, each patient was assigned to the highest risk category present among their detected genotypes.

### 2.7. Clinical Diagnosis Classification

Clinical diagnoses recorded at the time of HPV testing were obtained from the HIMS as ICD-10-coded entries. Because the case and control groups together presented with 68 distinct diagnostic labels, diagnoses were harmonised into five mutually exclusive clinical categories to enable meaningful between-group comparisons. The classification scheme was developed by the research team and applied identically to both groups.

Group 1 (Cervical Pathology) included mild, moderate, and unspecified cervical dysplasia, inflammatory disease of the uterine cervix, and abnormal uterine or vaginal bleeding. Group 2 (Anogenital and Viral Warts) comprised anogenital warts, viral warts, and dermatitis diagnoses with anogenital localisation. Group 3 (Vaginal and Vulvar Inflammation) encompassed acute vaginitis, vaginal inflammation, vulvovaginal candidiasis, bacterial vaginosis-related diagnoses, and inflammatory conditions of the vulva and perineum. Group 4 (Cystitis and Urinary Tract Infection) included acute cystitis, cystitis, urinary tract infection, urethritis, and functional disorders of the bladder. Group 5 (Other) incorporated all remaining diagnoses not assignable to Groups 1 through 4, including gynaecological screening visits, general examination, pelvic pain, endometriosis, ovarian cysts, menstrual disorders, male urological conditions (benign prostatic hyperplasia, chronic prostatitis, varicocele), and cases where no formal diagnosis was recorded. Given the clinical heterogeneity of Group 5, a sensitivity analysis was prespecified in which Group 5 was further divided into Group 5a (gynaecological screening and routine examinations, *n* = 27) and Group 5b (other unrelated clinical diagnoses, including endometriosis, ovarian cysts, menstrual disorders, benign prostatic hyperplasia, and abdominal pain, *n* = 63). Group 5a was used as the sole reference category in this sensitivity model.

### 2.8. Variable Coding and Data Preparation

All categorical variables were numerically coded prior to analysis. Sex was coded as female = 0 and male = 1. HPV test result was coded as negative = 0 and positive = 1. Geographic origin was coded as Turkish-born = 1 and foreign-born = 0. Risk category was coded as LR-HPV = 1, pHR-HPV = 2, and HR-HPV = 3. Co-infection status was defined as absence of co-infection (single genotype detected; coded 0) or presence of co-infection (two or more genotypes detected simultaneously; coded 1). Calendar month of testing was coded numerically from 1 (January) through 12 (December). Diagnosis group was coded as an ordinal variable (1 through 5) corresponding to the five categories described above. Patients with multiple HPV genotypes had their Cq values disaggregated into a long-format dataset in which each genotype contributed an individual row, while patient-level analyses retained a single Cq value per patient as reported by the LIS. For patient-level Cq analyses in co-infected individuals, the minimum Cq value across all detected genotypes was used as the patient-level viral load estimate, as this reflects the genotype with the highest viral copy number and therefore the greatest contribution to the overall infectious burden at the time of sampling. To assess the robustness of this selection criterion, a sensitivity analysis was conducted in which the Cq value of the first-listed genotype was substituted as an alternative patient-level estimate. The two approaches were highly correlated (Spearman rho = 0.917, *p* < 0.001), and no study conclusion was affected by the choice of method: the comparison between co-infected and single-genotype patients remained non-significant (minimum Cq: *p* = 0.836; first-listed Cq: *p* = 0.424), as did the comparison across risk categories (minimum Cq: *p* = 0.507; first-listed Cq: *p* = 0.507).

Given that specimen type was not recorded as a discrete variable in the institutional records, a further sensitivity analysis was conducted to assess whether the pooling of specimens from different anatomical sites influenced Cq comparisons. As male patients (*n* = 16) likely contributed penile or urethral swabs while female patients contributed cervical or vaginal specimens, all Cq analyses were repeated in the female-only subgroup (*n* = 81) as a proxy for anatomical site restriction. When restricted to female patients, the Kruskal–Wallis comparison across clinical diagnosis categories yielded H = 3.614, *p* = 0.461, and the post hoc comparison between Group 2 and Group 4 was no longer statistically significant (*p* = 0.057 uncorrected). These results indicate that the primary Cq finding is not fully robust to specimen-type heterogeneity and should be interpreted with caution.

### 2.9. Statistical Analysis

All statistical analyses were performed using GraphPad Prism (version 10.0, GraphPad Software, San Diego, CA, USA) as the primary platform, supplemented by IBM SPSS Statistics (version 29.0, IBM Corp., Armonk, NY, USA) for logistic regression modelling, R (version 4.3 or later; R Foundation for Statistical Computing, Vienna, Austria) for Cochran-Armitage trend testing, and Python (version 3.11 or later, with the NetworkX and SciPy libraries) for co-infection network analyses. A two-tailed significance threshold of alpha = 0.05 was applied throughout. For secondary and exploratory analyses involving multiple comparisons, the Bonferroni correction was applied, and the adjusted significance threshold (0.05 divided by the number of comparisons) is reported.

The normality of continuous variables (age and Cq values) was assessed using the Shapiro–Wilk test. Descriptive statistics for normally distributed continuous variables are presented as mean with standard deviation, while those for non-normally distributed variables are reported as median with interquartile range (IQR, 25th to 75th percentile). Categorical variables are expressed as absolute counts and proportions.

Comparisons between the HPV-positive and HPV-negative groups for continuous variables were performed using the independent samples *t*-test where normality was satisfied, or the Mann–Whitney U test otherwise. Categorical variables were compared using the chi-square test or Fisher’s exact test, as appropriate according to expected cell frequencies. For clinical diagnosis category comparisons, a pooled chi-square test across all five groups was reported alongside group-specific frequencies.

To identify independent predictors of HPV positivity, univariable binary logistic regression was first performed for each candidate predictor: age (continuous), sex (female as reference), geographic origin (Turkish-born as reference), and clinical diagnosis category (Group 5 as reference). Crude odds ratios (OR) and 95% confidence intervals (CI) were calculated. Variables meeting the pre-specified entry threshold of *p* less than 0.20 were entered into a multivariable logistic regression model using backward stepwise elimination. Overall model significance was assessed by the log-likelihood ratio test. Explained variance is reported as the Nagelkerke R-squared statistic.

Within the HPV-positive group, Cq values were compared across risk categories (three groups: HR, pHR, and LR), clinical diagnosis categories (five groups), co-infection status (two groups), and sex (two groups) using the Kruskal–Wallis test for comparisons involving three or more groups, with Bonferroni-corrected Dunn post hoc pairwise testing, or the Mann–Whitney U test for two-group comparisons. Associations between Cq values and age, and between Cq values and the number of co-infecting genotypes, were assessed using Spearman rank correlation.

Co-infection prevalence was reported as the proportion of HPV-positive patients with two or more simultaneously detected genotypes. Risk category composition within co-infection subgroups was compared using the chi-square test. The association between co-infection status and Cq value was evaluated using the Mann–Whitney U test, corresponding to the a priori hypothesis that the presence of multiple genotypes is not associated with higher relative viral copy number per swab.

Monthly HPV positivity rates were calculated as the number of HPV-positive tests divided by the total number of tests performed in each calendar month, multiplied by 100. The presence of a statistically significant directional trend in monthly positivity rates across the 12-month study period was assessed using the Cochran-Armitage trend test. May 2025 was excluded from this analysis due to the absence of testing records.

### 2.10. Ethical Considerations

This study was approved by the institutional ethics committee of Istanbul Atlas University prior to data collection and analysis (Protocol No: 05/16; Date: 11 May 2026). The study was classified as a retrospective archive review utilising routinely collected clinical and laboratory data, for which individual patient consent was waived in accordance with applicable national regulations governing non-interventional retrospective studies. The ethics approval date postdates the 2025 study period, which is consistent with the retrospective nature of the study: patient data from routine clinical care had already been recorded in institutional systems prior to the research protocol being defined. No data were extracted, analysed, or accessed prior to the receipt of ethics approval. The study therefore conforms to the standard ethical pathway for retrospective non-interventional archive reviews, in which approval is sought prior to data extraction rather than prior to the clinical care period from which records are drawn. All data were anonymised before analysis, and no patient-identifiable information was retained in the study dataset. The study was conducted in accordance with the principles of the Declaration of Helsinki and the applicable provisions of Turkish national research ethics guidelines.

## 3. Results

### 3.1. Baseline Characteristics

A total of 234 patients were included in the analysis, comprising 97 HPV-positive cases and 137 HPV-negative controls. The two groups were closely comparable with respect to demographic characteristics (Table 1). Mean age was 37.52 ± 9.78 years in the HPV-positive group and 37.91 ± 9.07 years in the HPV-negative group. As the Shapiro–Wilk test indicated a non-normal age distribution in the HPV-positive group (*p* = 0.039), the Mann–Whitney U test was used for between-group comparison, which showed no statistically significant difference (*p* = 0.662). The median age was 37.0 years in both groups, with interquartile ranges of 30.0 to 43.0 and 30.0 to 44.0, respectively, and overall age ranges of 21 to 60 years and 19 to 63 years.

The sex distribution was similarly balanced. Female patients constituted 83.5% of the HPV-positive group (*n* = 81) and 85.4% of the HPV-negative group (*n* = 117), with no significant difference between groups (chi-square test, *p* = 0.832). Regarding geographic origin, the vast majority of participants in both groups were Turkish-born patients, accounting for 92.8% of HPV-positive cases (*n* = 90) and 96.4% of HPV-negative controls (*n* = 132). Given the small expected cell counts, Fisher’s exact test was applied, and the difference in geographic origin was not statistically significant (*p* = 0.243).

In contrast, the distribution of clinical diagnosis categories differed significantly between the two groups (chi-square test, *p* = 0.015). Vaginal and vulvar inflammatory conditions (Group 3) represented the most frequent presentation in both groups, comprising 46.4% of HPV-positive and 33.6% of HPV-negative patients. Notably, anogenital and viral warts (Group 2) were substantially more common among HPV-positive patients (12.4%, *n* = 12) than HPV-negative controls (4.4%, *n* = 6). Conversely, the proportion of patients presenting for routine gynaecological screening or other non-specific complaints (Group 5) was markedly higher among HPV-negative patients (45.3%) compared to HPV-positive patients (28.9%), a pattern consistent with the clinical expectation that symptomatic presentations carry higher infection prevalence. Cervical pathology (Group 1) was uncommon and similarly represented in both groups (4.1% vs. 3.7%), while cystitis and urinary tract infection (Group 4) were proportionally more frequent among HPV-negative patients (13.1% vs. 8.2%).

### 3.2. HPV Genotype Distribution and Relative Viral Copy Number

Among the 97 HPV-positive patients, a total of 195 individual genotype detections were recorded across 32 distinct HPV types in the long-format dataset (Table 2). Following standard IARC classification [17], high-risk genotypes (HR-HPV; IARC Group 1), comprising HPV-16, 18, 31, 33, 35, 39, 45, 51, 52, 56, 58, 59, 66, and 68, collectively accounted for the largest proportion of detections, with a subtotal of 78 detections (40.00% of all genotype detections). Low-risk genotypes (LR-HPV; IARC Group 3) contributed the second highest absolute number of detections (*n* = 94, 48.21% of all genotype detections), a count that exceeds the number of HPV-positive patients (*n* = 97) owing to the contribution of patients harbouring multiple LR-type genotypes simultaneously. Probable and possible high-risk genotypes (pHR-HPV; IARC Groups 2A/2B), comprising HPV-53, 67, 70, 73, and 82, accounted for the smallest proportion of detections (*n* = 23, 11.79%). HPV-16 was the most frequently detected genotype overall and the most common HR type (*n* = 15, 7.69% of detections; 15.46% of patients), followed by HPV-6 (*n* = 19, 9.74% of patients) as the most frequently detected LR genotype.

Relative viral copy number per swab, approximated by Cq values (lower Cq indicating higher copy number), was numerically lowest (highest relative copy number) in the HR-HPV group (Mean Cq 19.81 ± 6.36; Median Cq 18.76, IQR: 15.19 to 25.57), compared to pHR-HPV (Mean Cq 22.67 ± 3.88; Median Cq 22.80, IQR: 20.69 to 25.32) and LR-HPV (Mean Cq 19.95 ± 6.64; Median Cq 21.83, IQR: 14.05 to 24.78), suggesting that high-risk genotypes may be associated with higher relative viral copy numbers at the time of sampling, though this interpretation requires caution given the absence of cellular DNA normalisation and the non-significant between-group difference (*p* = 0.507).

### 3.3. Co-Infection Patterns

Co-infection, defined as the simultaneous detection of two or more HPV genotypes, was identified in 43 patients (44.33%), while the remaining 54 patients (55.67%) harboured a single genotype (Table 3). Among patients with co-infection, the most common pattern involved two genotypes (*n* = 19, 19.59%), followed by three genotypes (*n* = 10, 10.31%), five genotypes (*n* = 6, 6.19%), four genotypes (*n* = 4, 4.12%), seven or more genotypes (*n* = 3, 3.09%), and six genotypes (*n* = 1, 1.03%).

Relative viral copy number, approximated by Cq values, did not differ significantly between patients with and without co-infection (Median Cq 22.73 vs. 20.89; Mann–Whitney U = 1050.5, *p* = 0.424), indicating that the presence of multiple genotypes was not associated with higher measurable viral copy number per swab. This is consistent with the interpretation that viral replication activity at the time of sampling is not proportional to the number of co-infecting genotypes. Mean ranks were 100.2 for the co-infection group and 88.69 for the single-genotype group, reflecting a non-significant trend rather than a true difference. Comparison of Cq values across co-infection subgroups (single genotype, HR-only, LR-only, and Mixed-risk) similarly yielded no significant differences (Kruskal–Wallis H = 1.107, *p* = 0.775).

The distribution of dominant IARC risk categories differed significantly according to co-infection status (χ^2^ = 18.82, df = 2, *p* = 0.0001). When each patient was classified according to their highest-risk genotype (dominant risk category), among patients without co-infection, LR-HPV was the predominant category (*n* = 28, 28.87%), followed by HR-HPV (*n* = 19, 19.59%) and pHR-HPV (*n* = 7, 7.22%). Among co-infected patients, mixed-risk co-infection, involving genotypes from more than one IARC risk category, constituted the most common pattern (*n* = 32, 32.99%), with HR-HPV as the dominant category in a further five patients (5.15%) and LR-HPV in seven patients (7.22%). Pure LR-HPV co-infection was identified in seven patients (7.22%) and pure HR-HPV co-infection in four patients (4.12%). No patient with co-infection carried exclusively pHR-HPV genotypes. These findings indicate that HR-HPV genotypes in this cohort were not restricted to single-genotype infections; four co-infected patients harboured exclusively HR-HPV types, and the majority of co-infected patients with an HR component presented with mixed-risk profiles involving concurrent lower-risk genotypes.

### 3.4. Relative Viral Copy Number Analysis

Cq values were compared across multiple clinically relevant subgroups within the HPV-positive cohort (Table 4). When patients were classified by their highest-risk genotype present according to standard IARC classification (patient-level risk category), Cq values did not differ significantly across the three risk groups (Kruskal–Wallis H = 1.360, *p* = 0.507). HR-HPV patients (*n* = 51) exhibited a mean Cq of 19.81 ± 6.36 (Median 18.76, IQR: 15.19 to 25.57), pHR-HPV patients (*n* = 11) a mean Cq of 22.67 ± 3.88 (Median 22.80, IQR: 20.69 to 25.32)**,** and LR-HPV patients (*n* = 35) a mean Cq of 19.95 ± 6.64 (Median 21.83, IQR: 14.05 to 24.78)**.** Pairwise post hoc comparisons after Bonferroni correction yielded no statistically significant differences (HR vs. pHR: *p* adjusted = 0.651; HR vs. LR: *p* adjusted = 1.000; pHR vs. LR: *p* adjusted = 1.000).

A statistically significant difference in Cq values was observed across clinical diagnosis categories (Kruskal–Wallis H = 10.519, *p* = 0.033). Post hoc analysis with Bonferroni correction identified a significant difference between Group 2 (Anogenital and Viral Warts; Mean Cq 16.08 ± 5.70) and Group 4 (Cystitis and Urinary Tract Infection; Mean Cq 24.24 ± 4.17), with an adjusted *p*-value of 0.011. This finding indicates that patients presenting with anogenital or viral warts harboured substantially higher relative viral copy numbers per swab compared to those with urinary tract complaints, which may reflect differences in the biological behaviour of HPV genotypes associated with these clinical presentations.

**Figure 1:**
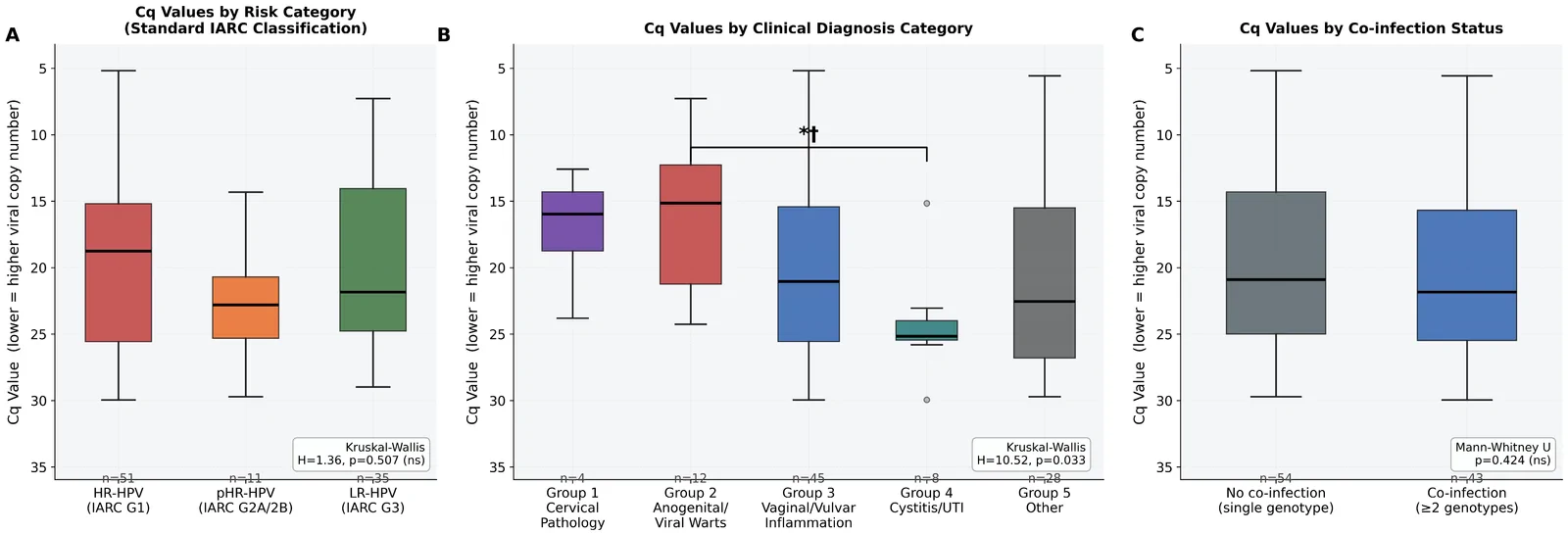
Box plots of Cq values across subgroups of HPV-positive patients (*n* = 97). (**A**) Cq values by HPV risk category under standard IARC classification: HR-HPV (IARC Group 1, *n* = 51), pHR-HPV (IARC Groups 2A/2B, *n* = 11), and LR-HPV (IARC Group 3, *n* = 35). Kruskal–Wallis H = 1.360, *p* = 0.507. (**B**) Kruskal–Wallis H = 10.52, *p* = 0.033; * denotes Bonferroni-corrected post hoc difference between Group 2 and Group 4 (*p*(adj) = 0.011); † this difference was non-significant when restricted to female patients (*p* = 0.057), reflecting specimen-site heterogeneity. (**C**) Mann–Whitney U, *p* = 0.424.

No significant differences in Cq values were found when patients were stratified by co-infection status (Mann–Whitney U, *p* = 0.424) or by sex (Mann–Whitney U, *p* = 0.528). Spearman rank correlation analyses revealed no significant associations between Cq values and patient age (rho = −0.045, *p* = 0.662) or the number of co-infecting genotypes (rho = −0.002, *p* = 0.983), further supporting the interpretation that relative viral copy number in this cohort was driven primarily by genotype-specific and clinical factors rather than demographic or co-infection-related variables. These findings are illustrated in Figure 1.

### 3.5. Predictors of HPV Positivity

Univariable logistic regression analyses were performed to identify potential predictors of HPV positivity across the full cohort (*n* = 234; Table 5). Age, sex, and geographic origin were not significantly associated with HPV status in univariable analysis (*p* = 0.753, *p* = 0.692, and *p* = 0.232, respectively). Among clinical diagnosis categories, Group 5 (Other) served as the reference. Two categories emerged as statistically significant predictors. Patients presenting with anogenital or viral warts (Group 2) had 4.43-fold higher odds of HPV positivity compared to the reference group (OR: 4.429, 95% CI: 1.509 to 12.999, *p* = 0.007). Patients with vaginal or vulvar inflammation (Group 3) similarly demonstrated significantly elevated odds of HPV positivity (OR: 2.166, 95% CI: 1.181 to 3.973, *p* = 0.013). No other diagnosis group reached statistical significance in univariable analysis.

To address the potential heterogeneity of Group 5, a prespecified sensitivity analysis was conducted using gynaecological screening visits (Group 5a, *n* = 27) as the sole reference category, thereby excluding patients with unrelated clinical diagnoses (Group 5b, *n* = 63; Table 5). The results were directionally consistent with the primary analysis and statistical significance was maintained for both Group 2 (anogenital and viral warts: OR 7.00, 95% CI: 1.84 to 26.61, *p* = 0.004) and Group 3 (vaginal and vulvar inflammation: OR 3.42, 95% CI: 1.27 to 9.27, *p* = 0.015). These findings confirm that the primary estimates were not an artefact of the heterogeneous reference group, and that patients presenting with HPV-related clinical features had consistently higher odds of positivity compared with those attending for routine gynaecological screening.

Both Group 2 and Group 3, meeting the pre-specified entry threshold of *p* less than 0.20, were entered into a multivariable logistic regression model using backward stepwise elimination (Table 6). Both variables retained statistical significance after mutual adjustment. Patients with anogenital or viral warts had 4.25-fold higher adjusted odds of HPV positivity compared to the reference group (aOR: 4.250, 95% CI: 1.488 to 12.140, *p* = 0.007). Patients with vaginal or vulvar inflammation had 2.08-fold higher adjusted odds (aOR: 2.079, 95% CI: 1.191 to 3.628, *p* = 0.010). The overall model was statistically significant (log-likelihood ratio test = 11.74, *p* = 0.003). The Nagelkerke R-squared value of 0.066 indicated modest explained variance, and the area under the receiver operating characteristic curve was 0.615, reflecting fair discriminatory ability. The model correctly classified 143 of 234 patients (61.1%).

### 3.6. Monthly Distribution and Temporal Trend

Monthly test volumes and HPV positivity rates across the 12-month study period are presented in Table 7. Overall, 234 patients were tested between January and December 2025. No HPV tests were recorded in May 2025 in either group, as no patients presented to the institution for HPV testing during this month. This likely reflects reduced healthcare utilisation during the extended public holiday period in early May in Türkiye. The absence of May records does not represent missing or lost data, and the Cochran-Armitage trend analysis was conducted across the eleven months for which patient data were available. March recorded the highest monthly test volume, accounting for 38 tests (16.2% of the annual total), of which 16 were HPV-positive (positivity rate 42.1%). In contrast, July had the smallest monthly sample (*n* = 9).

The highest monthly HPV positivity rates were recorded in June (63.6%), September (57.1%), and July (55.6%), suggesting a potential summer-to-early-autumn elevation in positivity. Conversely, the lowest positivity rate was observed in December (24.0%), with October and November also showing comparatively lower rates (33.3% and 36.0%, respectively). Despite this apparent seasonal pattern, the Cochran-Armitage trend test revealed no statistically significant directional trend in monthly positivity rates over the 12-month period (Z = −1.076, *p* = 0.282). This null result should be interpreted in the context of the relatively small and variable monthly sample sizes, which limit the statistical power to detect temporal trends within a single-year observation window.

Across the full study period, the overall HPV positivity rate was 41.5% (97 of 234 patients), a figure that is consistent with, and at the upper end of, positivity rates reported from tertiary care centres in Türkiye and neighbouring regions.

## 4. Discussion

The overall HPV positivity rate of 41.5% observed in the present study falls within the range reported from tertiary-level referral centres in Türkiye, where positivity estimates in clinically referred populations generally sit between 36% and 42%, and is substantially higher than figures from community-based screening cohorts [11]. A hospital-based study from Istanbul reported a comparable rate of 36.3%, closely approximating our findings [18]. This gap between referral-based and population-based estimates is most readily explained by the clinical make-up of tertiary-care cohorts. Patients who reach tertiary diagnostic platforms are more likely to be symptomatic, carry prior abnormal screening results, or present with established anogenital pathology, all of which increase the probability of a positive test. While this referral pattern limits the generalisability of our findings to the broader population, it is inherent to the study design, as the aim was not to reflect community-level prevalence but to examine HPV positivity patterns within a defined tertiary-care population.

Multivariable logistic regression identified clinical diagnosis category as the only independent predictor of HPV positivity; age, sex, and geographic origin were not significantly associated with infection status. Patients presenting with anogenital or viral warts had more than fourfold higher adjusted odds of testing positive, and those with vaginal or vulvar inflammatory conditions showed approximately twofold increased odds relative to patients attending for non-specific complaints or routine examinations. The model was statistically significant overall, correctly classified 61.1% of participants, and yielded a Nagelkerke R^2^ of 0.066 and an AUC of 0.615. The absence of significant associations for age and sex may appear surprising given that both are well-recognised predictors of HPV exposure and persistence in the broader literature [5,6,7]. However, this pattern is consistent with the epidemiological narrowing that characterises tertiary-care referral populations. In community-based cohorts, demographic variables reflect wide variation in sexual behaviour and exposure history; in a clinically selected population already enriched for suspected HPV-related disease, much of that variation is compressed. In this context, the clinical nature of the presentation—rather than who the patient is—becomes the more informative signal. That diagnosis category retained its predictive value after adjustment supports the view that symptom profile and disease phenotype independently influence HPV detection beyond what demographic factors alone can explain. A sensitivity analysis using gynaecological screening visits as the sole reference category confirmed that these associations were robust to the composition of the reference group.

The model’s explanatory power was, however, negligible. The Nagelkerke R^2^ of 0.066 indicates that the model accounts for only 6.6% of the variance in HPV positivity, leaving 93.4% unexplained. The McFadden pseudo R^2^ of 0.037 similarly reflects a poorly fitting model, and the AUC of 0.615 falls below the threshold of 0.70 that is conventionally regarded as acceptable discriminatory ability, indicating that the model performs only marginally better than chance classification. These metrics make clear that, while clinical diagnosis category reached statistical significance and the overall model was significant by the log-likelihood ratio test, statistical significance in this context should not be conflated with clinical predictive utility or causal primacy. The finding that clinical diagnosis category was the only significant predictor does not imply that it is the principal driver of HPV positivity; rather, it is the strongest signal available within the narrow range of variables accessible through routine institutional records. The vast majority of the unexplained variance almost certainly reflects behavioural, immunological, and sexual-history factors including number of lifetime sexual partners, condom use, smoking status, HPV vaccination history, and immune competence, none of which were consistently documented in the records available for this study and none of which could therefore be entered into the model. In this light, the appropriate interpretation is that clinical diagnosis category is a statistically detectable but weak and incomplete marker of HPV positivity within the constraints of retrospective administrative data, not that clinical presentation causally or predominantly determines HPV infection status.

Under standard IARC classification, low-risk genotypes (LR-HPV; IARC Group 3) remained the most frequently detected category (*n* = 94, 48.21% of all detections), followed by high-risk genotypes (HR-HPV; IARC Group 1; *n* = 78, 40.00%), while probable/possible high-risk genotypes (pHR-HPV; IARC Groups 2A/2B, comprising HPV-53, 67, 70, 73, and 82) contributed the smallest proportion (*n* = 23, 11.79%). Notably, the application of standard IARC criteria substantially increased the proportion of HR-HPV detections compared with our original classification, which had restricted this category to HPV-16 and HPV-18 alone. This pattern stands in contrast with the organised cervical screening cohorts, where high-risk oncogenic genotypes typically dominate the overall prevalence picture [10]. The likely explanation lies in the referral structure of the cohort: a large proportion of patients presented with anogenital warts or vulvovaginal inflammatory conditions, both of which are predominantly associated with low-risk HPV types [19,20]. The high proportion of low-risk detections therefore appears to reflect the clinical phenotype of the study population rather than any genuine regional epidemiological shift. That said, HPV-16 remained the dominant high-risk genotype (15.46% of patients), consistent with its well-documented prominence across multiple Turkish study populations [8,9,10]. Overall, these findings reinforce the point that genotype distributions in tertiary-care settings can look quite different from those in screening populations and should be read accordingly.

Co-infection with two or more HPV genotypes was found in 44.33% of HPV-positive patients, which exceeds rates commonly reported in large-scale screening populations [12] but is broadly in line with figures from tertiary referral and high-risk clinical cohorts. The elevated rate here is likely driven by two factors: the symptomatic nature of the study population, and the use of a broad-spectrum 37-genotype assay capable of picking up a wide range of concurrently present types. Risk category distribution differed significantly between single-genotype and co-infected patients, with mixed-risk co-infections—where genotypes from more than one risk category were present simultaneously—making up the most common pattern among co-infected patients (29.90%). Under the standard IARC classification, four patients (4.12%) harboured exclusively HR-HPV genotypes in co-infection, while mixed-risk co-infections, involving genotypes from more than one risk category, remained the dominant pattern among co-infected patients (*n* = 32, 32.99%). Pure LR-HPV co-infection was identified in seven patients (7.22%), and no patient carried exclusively pHR-HPV genotypes. The predominance of mixed-risk profiles suggests that oncogenic types in this cohort typically co-occurred with genotypes from other risk categories rather than accumulating in isolation, a pattern consistent with previous reports from clinically referred populations [11,12].

Quantitative viral load analysis showed no significant differences in Cq values across HPV risk categories, though there was a consistent numerical trend toward lower Cq values, reflecting higher relative viral copy number in patients harbouring high-risk genotypes. The most plausible explanation for the lack of significance is the considerable overlap in Cq distributions across risk categories, which likely reflects the heterogeneous mix of genotypes, lesion types, and viral activity states within each group rather than a true absence of between-category differences in viral kinetics. The directional trend nonetheless remains biologically coherent and aligns with previous work linking higher HR-HPV viral load to persistent infection and high-grade cervical lesions [13,14]. The clearest numerical difference in Cq values emerged across clinical diagnosis categories; with patients presenting with anogenital or viral warts showing lower Cq values than those with cystitis or urinary tract infection (Bonferroni-corrected post hoc *p* = 0.011). However, it must be acknowledged that Group 2 comprised predominantly male patients (*n* = 8/12, 66.7%) likely sampled from penile lesions, while Group 4 also included a high proportion of males (*n* = 5/8, 62.5%) likely sampled from urethral sites. When this analysis was restricted to female patients, the between-group difference was no longer statistically significant (*p* = 0.057). The biological interpretation that condylomatous lesions support high-level productive viral replication remains plausible, but cannot be disentangled from the contribution of specimen-site heterogeneity in the current dataset. This finding should therefore be regarded as exploratory and hypothesis-generating rather than confirmatory. By contrast, HPV detection in patients with urinary tract complaints likely reflects incidental or low-level carriage, with correspondingly lower relative viral copy number per swab. Cq values showed no significant associations with age, co-infection status, sex, or co-infection multiplicity, suggesting that relative viral copy number in this cohort was shaped primarily by the biological activity of infection at the tissue level rather than by demographic characteristics or the number of co-infecting genotypes.

Monthly positivity rates varied considerably throughout the study period, ranging from 24.0% in December to 63.6% in June, with the highest rates clustering in the summer and early autumn months. Despite this apparent pattern, the Cochran-Armitage trend test returned no significant directional trend over the 12-month observation window. This null result should be treated with caution: monthly test volumes fluctuated markedly—from as few as 9 in July to 38 in March—meaning that positivity estimates in low-volume months were inherently unstable. Beyond this, single-year retrospective datasets are poorly suited to characterising seasonal trends in HPV, where latency, persistence, and delayed clinical presentation all complicate temporal interpretation. Multi-year longitudinal data would be needed to determine whether the fluctuations observed here reflect true seasonal variation or simply random shifts in healthcare attendance and referral patterns.

The present findings should be interpreted in light of several methodological constraints. The retrospective single-centre design may limit the generalisability of the results to populations with similar referral characteristics and restrict the analysis to variables available within institutional record. Several key variables known to influence HPV acquisition, persistence, and clearance were unavailable in the institutional records and could not be included in the multivariable model, representing the most important source of residual confounding in the present study. HPV vaccination status was not consistently documented; given that national vaccination coverage in Türkiye remains suboptimal [16], the proportion of vaccinated individuals in the cohort is unknown, and differential vaccination rates between cases and controls could have introduced unmeasured protection bias. Smoking status was not recorded, despite its established role in modulating local cervical immunity and increasing susceptibility to persistent HPV infection. Sexual behaviour variables including number of lifetime and recent sexual partners, age at sexual debut, and condom use, were absent from the clinical records; these represent the most proximal behavioural determinants of HPV exposure and would be expected to contribute substantially to the 93.4% of unexplained variance in the multivariable model. Finally, immunosuppression status encompassing HIV infection, use of immunosuppressive medications, and other conditions impairing cell-mediated immunity, was not available as a discrete variable, despite being a well-established risk factor for HPV acquisition, persistence, and progression to high-grade disease. The absence of these four variable domains means that the multivariable model reflects only a fraction of the true aetiological complexity of HPV positivity, and the observed associations should be interpreted as hypothesis-generating signals rather than evidence of independent causal relationships. Lastly, Cq values offer only a relative viral copy number per swab and are subject to pre-analytical and platform-specific variability [21,22], which limits direct comparison with studies using different quantitative methods.

A further limitation concerns the heterogeneity of specimen types included in the analysis. Cervical, vaginal, and penile swab specimens were processed using the same qPCR platform and validated extraction system; however, anatomical sampling site was not recorded as a discrete variable in the institutional records, precluding formal adjustment for this factor. HPV viral load expressed as Cq values may not be directly comparable across different anatomical niches, as squamocolumnar junction, squamous epithelium, and urethral mucosa differ in cell density, viral tropism, and replication kinetics. Although the study assay incorporates an internal human control to verify extraction adequacy irrespective of specimen type, this does not fully account for site-specific differences in viral copy number per swab. In particular, the Cq comparison between clinical diagnosis groups was substantially influenced by the sex composition of those groups; Group 2 (anogenital warts) and Group 4 (cystitis/UTI) each contained a high proportion of male patients whose specimens likely originated from distinct anatomical sites. Sensitivity analyses restricted to female patients demonstrated that the post hoc difference between these groups was attenuated and no longer statistically significant, underscoring the need for caution in interpreting the Cq findings across diagnosis categories. The retrospective single-timepoint design does not permit distinction between transient HPV carriage and clinically persistent infection, which represents the biologically and clinically relevant endpoint for cervical carcinogenesis risk assessment. In the absence of longitudinal follow-up data, repeat testing at defined intervals, or histopathological correlation, the proportion of HPV-positive cases representing self-clearing incidental infections cannot be determined. The findings should therefore be interpreted as describing cross-sectional HPV positivity patterns within a tertiary-care referral population, rather than as indicators of viral persistence or disease progression risk. Prospective studies incorporating repeat testing and clinical follow-up are needed to determine whether the predictors of HPV positivity identified here also predict sustained infection. Finally, histopathological correlation was unavailable for most patients, making it impossible to assess lesion severity, persistence, or progression risk for the genotypes detected.

Despite these limitations, this study brings together demographic risk modelling, extended HPV genotyping, co-infection analysis, and quantitative viral load assessment within a single, well-characterised tertiary-care cohort. By drawing on molecular and clinical data from both female and male patients in a real-world diagnostic setting, it offers a multidimensional picture of HPV epidemiology relevant to tertiary referral practice in Türkiye [16]. The findings suggest that clinical context of presentation captures a statistically detectable but quantitatively modest signal within the complex multifactorial aetiology of HPV positivity in tertiary-care settings, and that genotype distribution and relative viral copy number data from such settings cannot be read directly through the lens of risk profiles derived from screening populations. Prospective studies incorporating behavioural and immunological covariates are needed to determine the true independent contribution of clinical presentation after full confounder adjustment.

## 5. Conclusions

The present study examined HPV positivity, genotype distribution, co-infection patterns, and quantitative viral load in a tertiary-care referral population using a 37-genotype real-time quantitative PCR platform. Across all analyses, the clinical context of presentation showed a statistically significant but quantitatively modest association with HPV positivity, and was a more consistent correlate of infection patterns than conventional demographic risk factors within the constraints of the available institutional data. It is important to emphasise that the study population was derived from a single tertiary referral centre and is therefore not representative of the general population. The observed HPV positivity rate of 41.5%, genotype distribution, and co-infection prevalence should not be interpreted as population-level prevalence estimates; these figures are likely influenced by referral bias, as patients reaching tertiary diagnostic platforms are substantially more likely to be symptomatic, carry prior abnormal results, or present with established anogenital pathology than unselected community members.

In multivariable analysis, clinical diagnosis category was the only independent predictor of HPV positivity. Patients presenting with anogenital warts or vulvovaginal inflammatory conditions carried significantly higher odds of testing positive, while age, sex, and geographic origin showed no meaningful associations. This likely reflects the epidemiological narrowing that occurs in tertiary-care settings, where patients are already selected for clinical suspicion of HPV-related disease, compressing the demographic variation that typically drives risk factor associations in community-based cohorts. The modest discriminatory capacity of the model suggests that unmeasured behavioural and immunological variables remain important contributors to positivity in this population.

The genotype distribution, dominated by high-risk and low-risk types, with HPV-16 leading among high-risk genotypes, mirrored the symptomatic composition of the cohort and did not represent a departure from established regional patterns. The co-infection rate of 44.33%, with mixed-risk profiles predominating and exclusively high-risk co-infections uncommon, suggests that oncogenic genotypes in this setting rarely occur in isolation. Relative viral copy number, as approximated by un-normalised Cq values, appeared more closely associated with clinical presentation category than with risk category, co-infection status, or patient demographics—a finding that, while exploratory given the absence of cellular DNA normalisation, suggests that the anatomical site and biological activity of the lesion at the time of sampling may be more informative determinants of measurable viral copy number than genotype risk category or demographic characteristics alone. These findings carry practical implications for how Cq-based data from routine diagnostic platforms should be interpreted in tertiary-care settings, where specimen heterogeneity and clinical diversity limit the comparability of un-normalised viral copy number estimates.

These results suggest that HPV diagnostic practice in tertiary-care settings should be guided by the clinical phenotype of presentation rather than risk profiles derived from screening populations alone. Extended genotyping paired with quantitative viral load assessment can provide meaningful diagnostic information beyond simple positivity, particularly in symptomatic patients and those with mixed-risk co-infections. The genotype distribution observed in this cohort, dominated by high-risk and low-risk types, with a high proportion of co-infections and mixed-risk profiles, may differ substantially from distributions reported in population-based cervical screening programmes, reflecting the clinical phenotype of a symptomatic referral population rather than community-level epidemiology. Clinicians and public health practitioners should exercise caution in extrapolating these findings beyond the tertiary-care referral context. Prospective studies incorporating behavioural risk data, vaccination history, and histopathological follow-up are needed to clarify the clinical significance and progression risk of the infection profiles described here.

## Figures and Tables

**Table 1:** Comparison of baseline demographic and clinical characteristics between HPV-positive and HPV-negative groups.

Variable	HPV-Positive (*n* = 97)	HPV-Negative (*n* = 137)	*p*-Value
Age			
Mean ± SD	37.52 ± 9.78	37.91 ± 9.07	0.662
Median (IQR)	37.0 (30.0–43.5)	37.0 (30.0–44.5)	
Range (min–max)	21–60	19–63	
Sex			
Female, *n* (%)	81 (83.51)	117 (85.40)	0.832
Male, *n* (%)	16 (16.49)	20 (14.60)	
Geographic Origin			
Turkish patient, *n* (%)	90 (92.78)	132 (96.35)	0.243
Foreign-born patient, *n* (%)	7 (7.22)	5 (3.65)	
Clinical Diagnosis Category			
Group 1: Cervical pathology, *n* (%)	4 (4.12)	5 (3.65)	**0.0150**
Group 2: Anogenital/Viral Warts	12 (12.37)	6 (4.38)	
Group 3: Vaginal/Vulvar inflammation, *n* (%)	45 (46.39)	46 (33.58)	
Group 4: Cystitis/Urinary tract infection, *n* (%)	8 (8.25)	18 (13.14)	
Group 5: Other, *n* (%)	28 (28.87)	62 (45.26)	

SD: Standard deviation; IQR: Interquartile range (25th–75th percentile). Bold *p*-values indicate statistical significance (*p* < 0.05). Group 1–5 sample sizes are consistent with the reference categories used in the univariable and multivariable logistic regression models (giving in following tables). Age was compared using the Mann–Whitney U test applied. Sex, geographic origin and clinical diagnosis category were compared by chi-square test.

**Table 2:** HPV genotype distribution and quantitative Cq values stratified by IARC risk category (*n* = 97 HPV-positive patients; 195 genotype detections).

HPV Type	*n* (%)	Mean Cq ± SD	Median Cq (IQR)
**High-Risk HPV (HR-HPV)—IARC Group 1 [Carcinogenic to humans]**			
HPV-16	15 (7.69)	19.03 ± 5.75	18.64 (14.96–24.71)
HPV-18	5 (2.56)	17.72 ± 7.32	15.61 (12.16–25.48)
HPV-31	7 (3.59)	18.38 ± 6.28	17.58 (14.81–24.21)
HPV-33	1 (0.51)	19.81	21.67
HPV-35	4 (2.05)	17.81 ± 2.25	17.88 (15.50–19.58)
HPV-39	4 (2.05)	20.05 ± 5.70	21.21 (14.81–25.06)
HPV-45	5 (2.56)	19.39 ± 5.03	18.78 (15.57–22.89)
HPV-51	5 (2.56)	21.97 ± 6.27	25.05 (16.61–27.55)
HPV-52	6 (3.08)	20.30 ± 5.76	20.49 (15.57–24.73)
HPV-56	8 (4.10)	21.08 ± 6.25	22.75 (15.24–27.25)
HPV-58	4 (2.05)	21.51 ± 4.93	22.11 (17.24–25.58)
HPV-59	6 (3.08)	22.70 ± 4.32	24.25 (19.75–25.42)
HPV-66	6 (3.08)	21.51 ± 6.23	23.72 (16.90–25.95)
HPV-68	2 (1.03)	20.83 ± 6.80	22.48 (14.29–26.96)
HR-HPV Subtotal: 78 detections (40.00%)			
**Probable/Possible High-Risk HPV (pHR-HPV)—IARC Group 2A/2B**			
HPV-53	9 (4.62)	20.29 ± 6.20	21.63 (16.20–24.97)
HPV-67	5 (2.56)	19.81 ± 4.57	20.88 (15.49–24.93)
HPV-70	1 (0.51)	22.27	22.27
HPV-73	5 (2.56)	23.68 ± 3.10	23.53 (20.64–26.73)
HPV-82	3 (1.54)	21.11 ± 4.99	23.07 (16.17–25.24)
pHR-HPV Subtotal: 23 detections (11.79%)			
**Low-Risk HPV (LR-HPV)—IARC Group 3** **[Not classifiable as carcinogen]**			
HPV-6	19 (9.74)	18.36 ± 5.90	19.54 (12.62–22.97)
HPV-11	6 (3.08)	21.10 ± 5.28	21.67 (17.02–25.12)
HPV-40	7 (3.59)	19.73 ± 6.28	20.08 (15.27–24.68)
HPV-42	12 (6.15)	21.66 ± 6.07	23.42 (17.98–26.47)
HPV-43	5 (2.56)	22.77 ± 6.31	23.89 (20.31–28.40)
HPV-44	3 (1.54)	17.45 ± 6.81	16.72 (12.64–24.82)
HPV-54	9 (4.62)	21.66 ± 4.55	22.80 (17.96–25.06)
HPV-61	3 (1.54)	26.76 ± 2.00	27.13 (24.71–28.45)
HPV-62	8 (4.10)	20.90 ± 6.18	22.92 (16.15–26.23)
HPV-72	4 (2.05)	20.58 ± 5.88	23.29 (14.74–25.11)
HPV-74	5 (2.56)	22.84 ± 4.57	23.53 (17.96–26.09)
HPV-84	2 (1.03)	22.45 ± 6.23	25.06 (17.69–26.60)
HPV-91	7 (3.59)	23.62 ± 3.66	22.93 (21.29–27.58)
LR-HPV Subtotal: 94 detections (48.21%)			

Cq: Cycle quantification (amplification threshold); lower Cq = higher viral copy number. IQR: Interquartile range (25th–75th percentile). HR: High-Risk; pHR: Probable/Possible High-Risk (IARC Group 2A/2B); LR: Low-Risk (IARC Group 3). IARC: International Agency for Research on Cancer. Individual genotype detection frequencies expressed as percentages of patients (*n* = 97) may exceed 100% in aggregate within and across risk categories, because patients harbouring multiple genotypes (co-infections) contribute one detection per genotype. For example, a patient carrying three HPV types simultaneously contributes to three separate genotype frequency counts. Subtotal and Grand Total percentages are calculated relative to the total number of genotype detections in the long-format dataset (*n* = 195) and sum to 100% across risk categories.

**Table 3:** Co-infection prevalence, quantitative Cq values and dominant risk category distribution by number and risk pattern of co-infecting HPV genotypes.

Co-infection Group	*n* (%)	Mean Cq ± SD	Median Cq (IQR)	*p* ^†^	Dominant Risk Category, *n* (%)	*p* ^‡^
No co-infection (single genotype)	54 (55.67)	19.72 ± 6.65	20.89 (14.14–25.24)	U = 1050.5 *p* = 0.424	19 (19.59%)/ 7 (7.22%)/ 28 (28.87%)	χ^2^ = 18.82, df = 2, ***p* = 0.0001**
Co-infection (≥2 genotypes)	43 (44.33)	21.16 ± 5.71	22.73 (16.67–25.56)		32 (32.99%)/ 15 (15.46%)/ 7 (7.22%)	
**Subgroups**	***n* (%)**	**Mean Cq ± SD**	**Median Cq (IQR)**	*p* *	**HR/pHR/LR, *n* (%)**	
HR-HPV only co-infection	4 (4.12%)	21.24 ± 5.30	21.13 (16.48–25.89)	H = 1.107, *p* = 0.775	—	—
pHR-HPV only co-infection	0 (0.00%)	—	—		—	
LR-HPV only co-infection	7 (7.22%)	22.38 ± 5.43	23.35 (22.32–24.92)		—	
Mixed-risk co-infection (HR+pHR and/or LR)	32 (32.99%)	20.36 ± 5.95	19.78 (15.56–25.45)		—	

^†^ Cq comparison (co-infection vs. no co-infection ≥ 2 genotypes): Mann–Whitney U test (U = 1050.5, *p* = 0.424); Mean Ranks: No co-infection = 88.69, Co-infection = 100.2. Kruskal–Wallis test across co-infection subgroups (single, HR-only, LR-only, Mixed): H = 1.107, *p* = 0.775. ^‡^ Dominant risk category distribution comparison (no co-infection vs. co-infection): chi-square test (χ^2^ = 18.82, df = 2, *p* = 0.0001). In summary rows (No co-infection/Co-infection ≥ 2 genotypes), HR/pHR/LR values reflect the dominant (highest) IARC risk category per patient; each patient is counted once and values sum to group totals. * HR/pHR/LR breakdown is not reported for co-infection subgroup rows (HR-only, pHR-only, LR-only, Mixed-risk) as risk composition is implicit in the subgroup definition. For mixed-risk co-infection, patients by definition harbour genotypes from more than one risk category simultaneously, precluding a single-category assignment. Cq: Cycle quantification (amplification threshold); lower Cq = higher relative viral copy number per swab. IQR: Interquartile range (25th–75th percentile). HR: High-Risk (IARC Group 1); pHR: Probable/Possible High-Risk (IARC Group 2A/2B); LR: Low-Risk (IARC Group 3). HPV-83 classified as LR-HPV. Bold *p*-values indicate statistical significance (*p* < 0.05).

**Table 4:** Comparison of Cq values across risk categories (HR/pHR/LR), clinical diagnosis groups, co-infection status and sex (HPV-positive group *n* = 97).

Subgroups					
**By Risk Category** **(Standard IARC classification)**	***n* (%)**	**Mean Cq ± SD**	**Median Cq (IQR)**	**Range**	*p* *
HR-HPV	51 (52.58)	19.81 ± 6.36	18.76 (15.19–25.57)	5.18–29.95	0.507
pHR-HPV	11 (11.34)	22.67 ± 3.88	22.80 (20.69–25.32)	14.31–29.71	
LR-HPV	35 (36.08)	19.95 ± 6.64	21.83 (14.05–24.78)	7.27–28.98	
Post hoc (Bonferroni k = 3): HR vs. pHR *p*(adj) = 0.651; HR vs. LR *p*(adj) = 1.000; pHR vs. LR *p*(adj) = 1.000					
**By Clinical Diagnosis Category**	***n* (%)**	**Mean Cq ± SD**	**Median Cq (IQR)**	**Range**	*p* *
Group 1: Cervical pathology	4 (4.12)	17.08 ± 4.84	15.96 (14.30–18.74)	12.58–23.80	**0.0325**
Group 2: Anogenital/Viral Warts	12 (12.37)	16.08 ± 5.95	15.14 (12.27–21.24)	7.27–24.26	
Group 3: Vaginal/Vulvar inflammation	45 (46.39)	20.23 ± 6.29	21.03 (15.42–25.56)	5.18–29.95	
Group 4: Cystitis/Urinary tract infection	8 (8.25)	24.24 ± 4.17	25.16 (23.99–25.46)	15.16–29.95	
Group 5: Other	28 (28.87)	21.16 ± 6.46	22.54 (15.50–26.81)	5.57–29.71	
Post hoc (Bonferroni k = 10): Group 2 vs. Group 4: *p*(adj) = 0.011; All other pairs: *p*(adj) > 0.05					
**By Co-infection Status**	***n* (%)**	**Mean Cq ± SD**	**Median Cq (IQR)**	**Range**	*p* *
No co-infection (single genotype)	54 (55.67)	19.72 ± 6.65	20.89 (14.31–25.00)	5.18–29.71	0.424
Co-infection (≥2 genotypes)	43 (44.33)	20.77 ± 5.93	21.83 (15.67–25.48)	5.57–29.95	
**By Sex**	***n* (%)**	**Mean Cq ± SD**	**Median Cq (IQR)**	**Range**	*p* *
Female	81 (83.51)	20.39 ± 6.15	21.03 (15.42–25.34)	5.18–29.95	0.528
Male	16 (16.49)	19.16 ± 7.31	21.76 (12.64–24.58)	7.27–29.95	

* *p* < 0.05. Risk category and clinical diagnosis: Kruskal–Wallis test with Bonferroni-corrected Dunn post hoc. Sex and co-infection status: Mann–Whitney U test. Spearman correlation used for Cq vs. age and Cq vs. number of co-infecting genotypes. Bold *p*-values indicate statistical significance (*p* < 0.05).

**Table 5:** Univariable binary logistic regression analysis of predictors of HPV positivity with sensitivity analysis using gynaecological screening as reference (Group 5a).

Variable	β	Crude OR	95% CI	*p*
Age (continuous)				
Age (per 1-year increment)	−0.004	0.996	0.968–1.024	0.753
Sex				
Female (reference)	Ref.	1.000	—	—
Male	+0.145	1.156	0.565–2.364	0.692
Geographic Origin				
Turkish-born (reference)	Ref.	1.000	—	—
Foreign-born	+0.719	2.053	0.632–6.672	0.232
Clinical Diagnosis Category				
Group 5—Other (reference)	Ref.	1.000	—	—
Group 1: Cervical pathology, *n* (%)	+0.572	1.771	0.442–7.101	0.420
Group 2: Anogenital/Viral Warts	+1.488	4.429	1.509–12.999	**0.007**
Group 3: Vaginal/Vulvar inflammation, *n* (%)	+0.773	2.166	1.181–3.973	0.013
Group 4: Cystitis/Urinary tract infection, *n* (%)	−0.016	0.984	0.383–2.532	0.974
Sensitivity analysis *				
Group 5a—Gynaecological screening	Ref.	1.000	—	—
Group 1—Cervical pathology	+1.030	2.800	0.567–13.833	0.207
Group 2—Anogenital/Viral Warts	+1.946	7.000	1.841–26.613	**0.004**
Group 3—Vaginal/Vulvar inflammation	+1.231	3.424	1.265–9.270	**0.015**
Group 4—Cystitis/UTI	+0.442	1.556	0.454–5.330	0.482

OR: Odds Ratio; CI: Confidence Interval; β: regression coefficient. Diagnosis group sample sizes; Group 1: *n* = 9 (HPV+4/HPV-5, 3.8%); Group 2: *n*=18 (HPV+12/HPV-6, 7.7%); Group 3: *n* = 91 (HPV+45/HPV-46, 38.9%); Group 4: *n* = 26 (HPV+8/HPV-18, 11.1%); Group 5: *n* = 90 (HPV+28/HPV-62, 38.5%). Variables with *p* < 0.20 entering multivariable model: Group 2—Anogenital/Viral Warts (*p* = 0.0068) and Group 3—Vaginal/Vulvar inflammation (*p* = 0.0125). Dependent variable: HPV status (0 = Negative, 1 = Positive). *n* = 234 (HPV-positive = 97, HPV-negative = 137). Variables with *p* < 0.20 entered multivariable model. * Group 5a: gynaecological screening visits and routine examinations (*n* = 27: HPV-positive = 6, HPV-negative = 21). Group 5b (other clinical diagnoses without HPV-related presentation, *n* = 63) excluded from this analysis. Model fit: Nagelkerke R^2^ = 0.093, AUC = 0.639. Bold *p*-values indicate statistical significance (*p* < 0.05).

**Table 6:** Multivariable logistic regression: independent predictors of HPV positivity.

Variable	β	Adjusted OR (aOR)	95% CI	*p*
(Variables with *p* < 0.20 in univariable analysis will be entered into the model)				
Group 5—Other (reference)	Ref.	1.000	—	—
Group 2—Anogenital/Viral Warts	+1.447	4.250	1.488–12.140	**0.007**
Group 3—Vaginal/Vulvar inflammation	+0.732	2.079	1.191–3.628	**0.010**
Model Fit Statistics				
Sample size		*n* = 234 (HPV-positive = 97/HPV-negative = 137)		
Overall model significance (LLR test)		χ^2^ = 11.74, *p* = 0.003		
McFadden Pseudo R^2^		0.0370		
Nagelkerke R^2^		0.0659		
AUC (ROC curve)		0.6146		
Correctly classified		143/234 (61.1%)		

aOR: Adjusted Odds Ratio; AUC: Area Under ROC Curve; LLR: Log-Likelihood Ratio test. Backward stepwise elimination. Dependent variable: HPV status (0 = Negative, 1 = Positive). Reference category: Group 5—Other. Two variables with *p* < 0.20 in univariable analysis were entered (Groups 2 and 3). Both remained significant in the final model. Bold *p*-values indicate statistical significance (*p* < 0.05).

**Table 7:** Monthly HPV test volumes, positivity counts and HPV positivity rates across the 12-month study period.

Month	HPV Positive *n* (%)	HPV Negative *n* (%)	Total *n* (%)	HPV Positivity Rate (%)	*p* *
January	6 (6.2%)	11 (8.0%)	17 (7.3%)	35.3%	0.282
February	9 (9.3%)	10 (7.3%)	19 (8.1%)	47.4%	
March	16 (16.5%)	22 (16.1%)	38 (16.2%)	42.1%	
April	9 (9.3%)	12 (8.8%)	21 (9.0%)	42.9%	
June	7 (7.2%)	4 (2.9%)	11 (4.7%)	63.6%	
July	5 (5.2%)	4 (2.9%)	9 (3.8%)	55.6%	
August	10 (10.3%)	14 (10.2%)	24 (10.3%)	41.7%	
September	12 (12.4%)	9 (6.6%)	21 (9.0%)	57.1%	
October	8 (8.2%)	16 (11.7%)	24 (10.3%)	33.3%	
November	9 (9.3%)	16 (11.7%)	25 (10.7%)	36.0%	
December	6 (6.2%)	19 (13.9%)	25 (10.7%)	24.0%	
Total	97 (41.5%)	137 (58.5%)	234 (100%)	41.5%	

* Cochran-Armitage trend test for linear trend in monthly HPV positivity rates over the 12-month period: Z = −1.076, *p* = 0.2818 (ns). No statistically significant directional trend was detected across the study year. The *p*-value is shown in the first data row for reference. ns: not significant (*p* > 0.05). May excluded from trend analysis due to no HPV records during the month.

## Data Availability

The data presented in this study are available on request from the corresponding author due to privacy restrictions.

## Abbreviations

The following abbreviations are used in this manuscript:

HPV	Human Papillomavirus
qPCR	Quantitative Polymerase Chain Reaction
RT-qPCR	Real-Time Quantitative Polymerase Chain Reaction
Cq	Cycle Quantification
NAT	Nucleic Acid Testing
IC	Internal Control
NTC	Negative Control
PC	Positive Control
LoD	Limit of Detection
IARC	International Agency for Research on Cancer
HR-HPV	High-Risk HPV
pHR-HPV	Possible High-Risk HPV (also referred to as pHR, Probable High-Risk)
LR-HPV	Low-Risk HPV
OR	Odds Ratio
aOR	Adjusted Odds Ratio
CI	Confidence Interval
AUC	Area Under the Receiver Operating Characteristic Curve
ROC	Receiver Operating Characteristic
SD	Standard Deviation
IQR	Interquartile Range
UTI	Urinary Tract Infection
LIS	Laboratory Information System
HIMS	Hospital Information Management System
IRB	Institutional Review Board
EPV	Events Per Variable
